# Evaluation of effortful swallow in patients with post stroke dysphagia using visual analysis of swallowing efficiency and safety (VASES) protocol

**DOI:** 10.1007/s00405-025-09920-w

**Published:** 2026-03-26

**Authors:** Mohammed Ali Saad Baraka, Yossra Abdel Naby Sallam, Hedia Muhey ElNeshwey, Hend Abdel Fattah Emam

**Affiliations:** 1 Present Address: Phoniatrics Unit, Otorhinolaryngology Department, Faculty of Medicine, Ain Shams University, Cairo, Egypt; 2 Phoniatrics Unit, Otorhinolaryngology Department, Faculty of Medicine for Girls, Al-Azhar University, Cairo, Egypt

**Keywords:** Dysphagia, Effortful swallow, Stroke, VASES, FEES

## Abstract

**Background:**

The effortful swallow (ES) maneuver is widely used in clinical practice, designed to enhance the safety and efficiency of swallowing. Despite its common use in clinical practice, there have been relatively few research studies exploring the impact of effortful swallowing in clinical populations with dysphagia.

**Objective:**

To evaluate the application of the ES maneuver in post-stroke patients with dysphagia through a structured, protocol-based approach utilizing the Visual Analysis of Swallowing Efficiency and Safety tool (VASES).

**Materials and methods:**

This cross-sectional study involved 34 adults with poststroke dysphagia. Each patient underwent a flexible endoscopic evaluation of swallowing (FEES) in the dysphagia clinic of a tertiary care hospital’s phoniatrics unit. An ES maneuver was incorporated into the FEES procedure. The VASES protocol was employed to systematically assess the FEES results. The effect of the ES maneuver was quantified by calculating Cohen’s d for pre- and post-maneuver measurements from the VASE protocol.

**Results:**

Largest residue reductions were found in 10 cc of thin fluids (oropharyngeal: pre-ES d = 1.32, post-ES effect size d = 1.57; hypopharyngeal: pre-ES d = 1.39, and post-ES effect size d = 1.93).

**Conclusion:**

The application of an ES maneuver significantly improves pharyngeal clearance and airway protection for larger and more complex bolus volumes.

## Introduction

Oropharyngeal dysphagia (OD) refers to the perceived or actual difficulty in safely forming or transporting a bolus from the oral cavity to the stomach, and it is considered a symptom rather than a standalone disease. This condition affects between 10% and 33% of older adults, with a particularly high prevalence among patients immediately after stroke [1]. Dysphagia following a stroke is a common complication that significantly influences patient recovery and overall quality of life, as approximately 50% of stroke survivors experience swallowing difficulties, which can lead to serious health issues such as malnutrition, dehydration, and an increased risk of aspiration pneumonia [2].

A range of clinical and instrumental diagnostic methods are employed to diagnose and monitor patients with dysphagia [3]. Fiberoptic endoscopic evaluation of swallowing (FEES) and video fluoroscopy (VFSS) are regarded as the gold standards for assessing dysphagia and detecting pharyngeal residue [4]. Among these, FEES has emerged as the preferred assessment technique for both phoniatricians and otorhinolaryngologists. Its growing popularity can be attributed to its effectiveness, safety, affordability, broad applicability, and suitability for various clinical settings [5].

Current FEES scoring protocols principally quantify three pathological findings: (1) premature bolus spillage into the pharynx, (2) laryngeal penetration/aspiration, and (3) residual material post-swallow [5]. Clinicians exhibit considerable variability in their interpretation of FEES. Several scales have been developed to standardize FEES [6], including the Penetration Aspiration Scale [7], Yale Pharyngeal Residue Severity Rating Scale [8], the Pooling Score [9], the Boston Residue and Clearance Scale [10], the Mansoura Fiberoptic Endoscopic Evaluation of Swallowing Residue Rating Scale [11], and Dynamic Imaging Grade of Swallowing Toxicity [12]. Although all these scales utilize categorical ratings, they differ significantly in terms of the outcomes measured, the number of categories, and their definitions. Furthermore, categorical methods for FEES may be less reliable or sensitive than visual analogue scales in detecting subtle differences [13].

The Visual Analysis of Swallowing Efficiency and Safety (VASES) protocol is an established, transparent, reliable, and standardized rating method for FEES. It creates clearly defined anatomic and temporal boundaries and uses 100-point measures rather than categorical measures to judge functional swallowing impairments [14].

Dysphagia treatment targets specific physiological swallowing deficits that can lead to airway invasion and residue, necessitating the selection of rehabilitative and compensatory strategies tailored to the affected skills. Compensatory techniques, such as adjusting head positions and modifying bolus consistency, help reduce immediate swallowing risks by altering bolus flow or timing but do not change the underlying swallowing physiology [15]. In contrast, rehabilitative strategies aim to strengthen the muscles involved in swallowing and promote long-lasting physiological changes. The effortful swallow maneuver serves both as a compensatory method to enhance bolus flow through the pharynx and as a rehabilitative technique that modifies physiological aspects of swallowing [16].

The effortful swallow (ES) is widely used in clinical practice due to its recommended role in enhancing swallowing safety and efficiency. Despite its use, the application of ES in dysphagic populations remains under-researched. A systematic review by Bahia and Lowell examined the physiological effects of the effortful swallow maneuver and found that the quality assessment of each study depends on three variables: effect size measurements, reliability of outcome reporting, and the use of blinded assessments of outcomes. To accurately evaluate the effects of the ES on swallowing safety and efficiency, it is essential to study individuals with swallowing disorders using instrumental evaluations [17]. Most studies have focused on normal populations with a small number of participants [17], which limits the generalizability of the findings. While many studies have used videofluoroscopy to assess its effectiveness, this method may not be available in all settings, particularly in tertiary care facilities.

Furthermore, inconsistent outcomes across studies may arise from a lack of standardized instructions for performing the ES [17]. Therefore, this study aimed to evaluate the immediate effect of the ES maneuver in a broad post-stroke cohort with dysphagia using the VASES protocol, a tool designed to quantify swallowing safety and efficiency across controlled volumes and consistencies during FEES.

##  Study design

- This cross-sectional study was conducted from May 2024 to June 2025, involving 34 adult post-stroke patients with dysphagia at the Phoniatrics Units of the Otorhinolaryngology Departments in Cairo’s tertiary medical centers, specifically Ain Shams and Al Azhar University Hospitals.

## Subjects

Inclusion criteria included:

- Adult patients, both males and females (≥ 18 years) with a confirmed stroke.
- Referred to the dysphagia clinic as part of routine pre-discharge care.
- 2–3 weeks after their stroke, following improvements in their overall condition.
- Presence of pharyngeal residue confirmed via a Flexible Endoscopic Evaluation of Swallowing (FEES) examination.

Exclusion criteria included: cognitive impairment, the use of head stabilization devices or other physical limitations, inability to follow simple instructions, intolerance to FEES, bilateral nasal obstruction, or refractory epistaxis.

The Ain Shams Institute’s Ethical Committee of Human Research (reference number: FWA000017585) approved this research; the approval number is FMASU MS422/2024.

## Sample size

The PASS 15 program was used to determine the sample size, setting power at 80% and alpha error at 0.05. It is estimated that a sample size of 34 patients will be enough.

## Study procedure

All participants underwent the following procedures:

1-A patient interview and comprehensive history taking, along with general, neurological, and vocal tract examinations.

2-The Arabic Eating Assessment Tool-10 (A-EAT-10) [18]:

The subjective assessment of dysphagia was performed using the validated A-EAT-10. This instrument consists of 10 questions where patients rate their swallowing difficulty on a scale from 0 (never) to 4 (always). The total score is the sum of all items, with a score of 3 or higher indicating the presence of oropharyngeal dysphagia, while scores below 3 are considered normal. Selected patients were given the validated A-EAT-10. The tool was explained to them, and for illiterate patients, their caregivers read the questions and recorded the responses.

3-An instrumental swallowing evaluation utilizing fiberoptic endoscopic evaluation of swallowing (FEES), the assessment protocol is divided into three stages: The first stage involves a thorough and careful observation of the anatomy, secretions, and the movements of the nasal structures when the patient is asked to speak and breathe. The second stage involves evaluating patients for swallowing by administering a single, non-repeated sequence of consistencies: thin liquids (1, 3, 5, and 10 cc of water), a teaspoon of semisolid (yogurt), and solid (a small piece of bread). Green food coloring was added to all materials to improve endoscopic visibility. While the protocol typically started with thin liquids, the initial consistency was tailored to the patient’s history, resulting in two patients beginning with the semisolid. In the third stage, Patients were instructed to perform an ES. Successful patient performance of the maneuver was facilitated by examiner’s demonstration and verbal cues during FEES. Given the absence of prior research on the ES in the Arab world, we developed a culturally appropriate instruction in Arabic. The prompt was:

“اضغط بلسانك على سقف حلقك وابلع بقوة، كأن هناك لقمة جافة عالقة في حلقك، وتحاول دفعها.”

“Press your tongue against the roof of your mouth and swallow hard, as if there is a dry piece of food stuck in your throat, and you are trying to push it down”. This was to be performed once with every consistency and volume tested.

-After completing the FEES procedure and recording the videos, three phoniatricians worked independently, with each completing their evaluation in a single, dedicated session (each rater completed her entire assessment within one session) using the VASES protocol to assess inter-rater reliability. Furthermore, intra-rater reliability was assessed through repeated measurements conducted by a single observer in the same setting over two weeks.

**Fig. 1:**
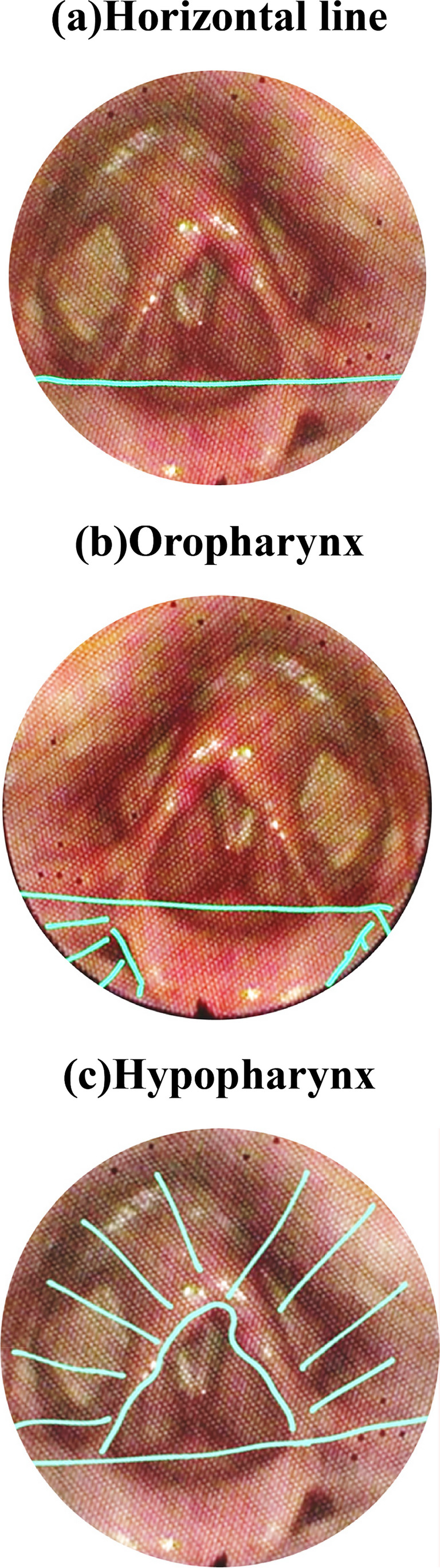
Oropharynx and hypopharynx anatomic boundaries. Horizontal line (**a**) Oropharynx (**b**) Hypopharynx (**c**)

**Fig. 2:**
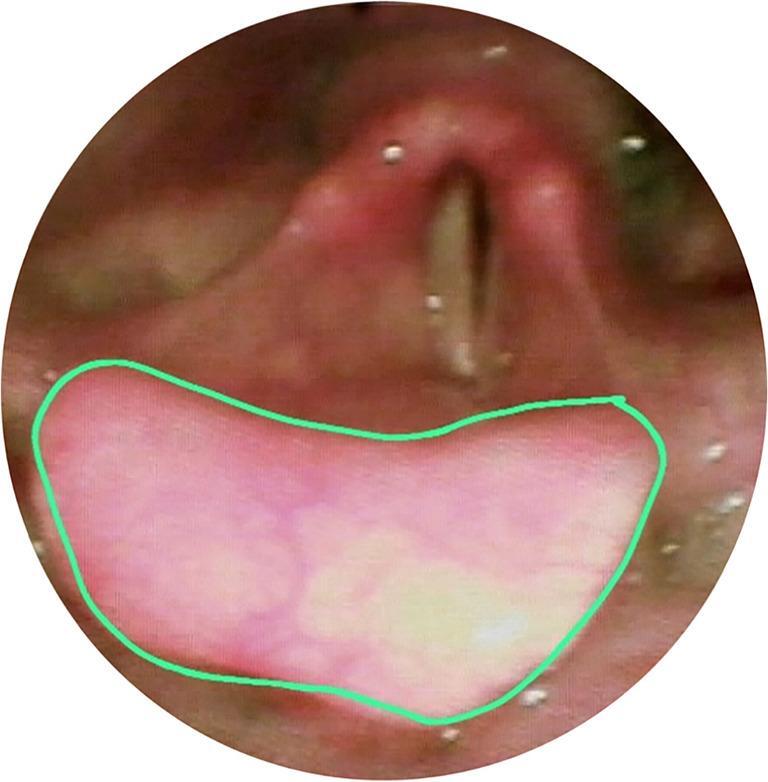
Anatomical Boundaries of the Laryngeal Surface of the Epiglottis

**Fig. 3:**
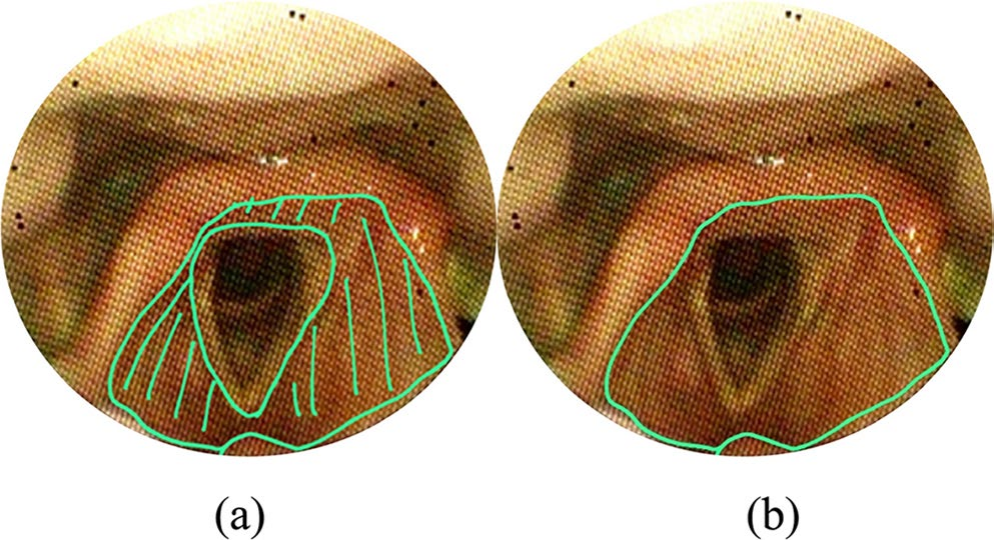
Anatomical boundaries of the laryngeal vestibule

**Fig. 4:**
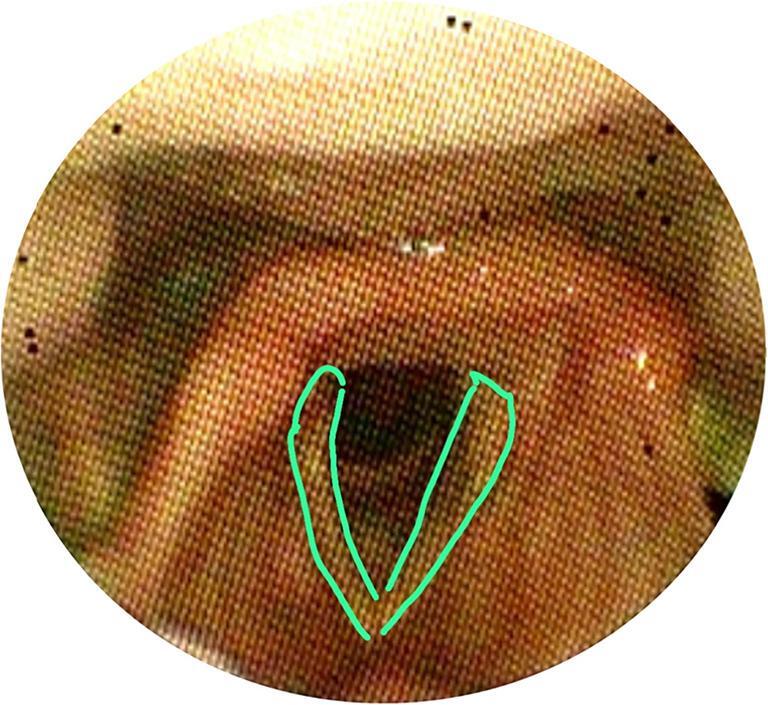
Anatomical boundaries of the vocal folds

**Fig. 5:**
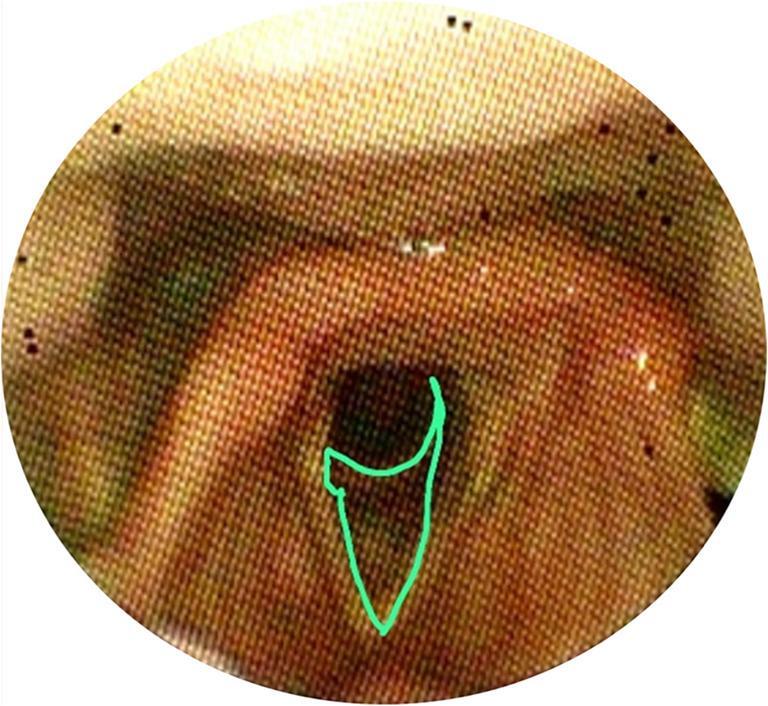
Subglottis anatomic boundary

The VASES protocol [14] employs a 100-point visual analog scale to measure the extent of residue coverage on specific anatomical structures, including the oropharynx, hypopharynx, epiglottis, laryngeal vestibule, vocal folds, subglottis, and penetration and aspiration scale (PAS), based on clearly defined anatomical boundaries. Scores range from 0% (indicating no residue) to 100% (indicating complete coverage). The anatomical divisions are as follows: The oropharynx and hypopharynx as seen in are divided by a horizontal line at the junction of the aryepiglottic folds and the epiglottis as seen in figure (1a). The oropharynx lies above and in front of this line shown in figure (1b), while the hypopharynx is below and behind it figure (1c). This definition excludes the laryngeal vestibule and the epiglottis itself.; the surface of the epiglottis is defined above a curved line from the aryepiglottic folds to the epiglottic trough shown in Figure (2); the laryngeal vestibule lies below the epiglottic surface bordered by the aryepiglottic folds and arytenoids in Figures (3a & 3b); the vocal folds include the ventricles, full fold surfaces, and cartilaginous parts in Figure (4) and the subglottis extends from below the vocal folds down to the cricoid and trachea see in Figure (5). If residue coats the mucosa without any visible pooling, it is rated as 3% or less. Only residues that appear new and correspond in color and consistency to the bolus under evaluation are considered. In instances where residue is mixed with secretions, only the residue should be estimated, ignoring any secretions.

**Fig. 6:**
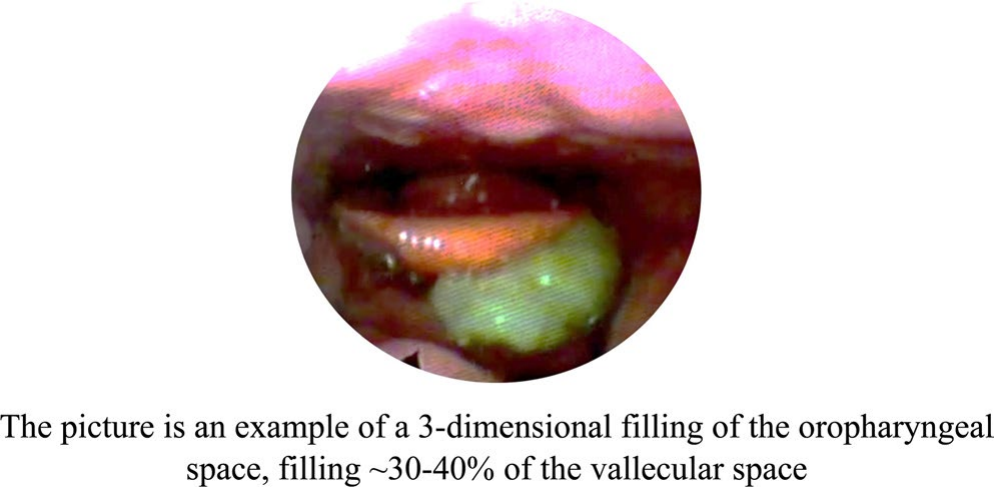
Example of a 3-dimensional filling of the oropharyngeal space, filling ~30-40% of the vallecular space

**Fig. 7:**
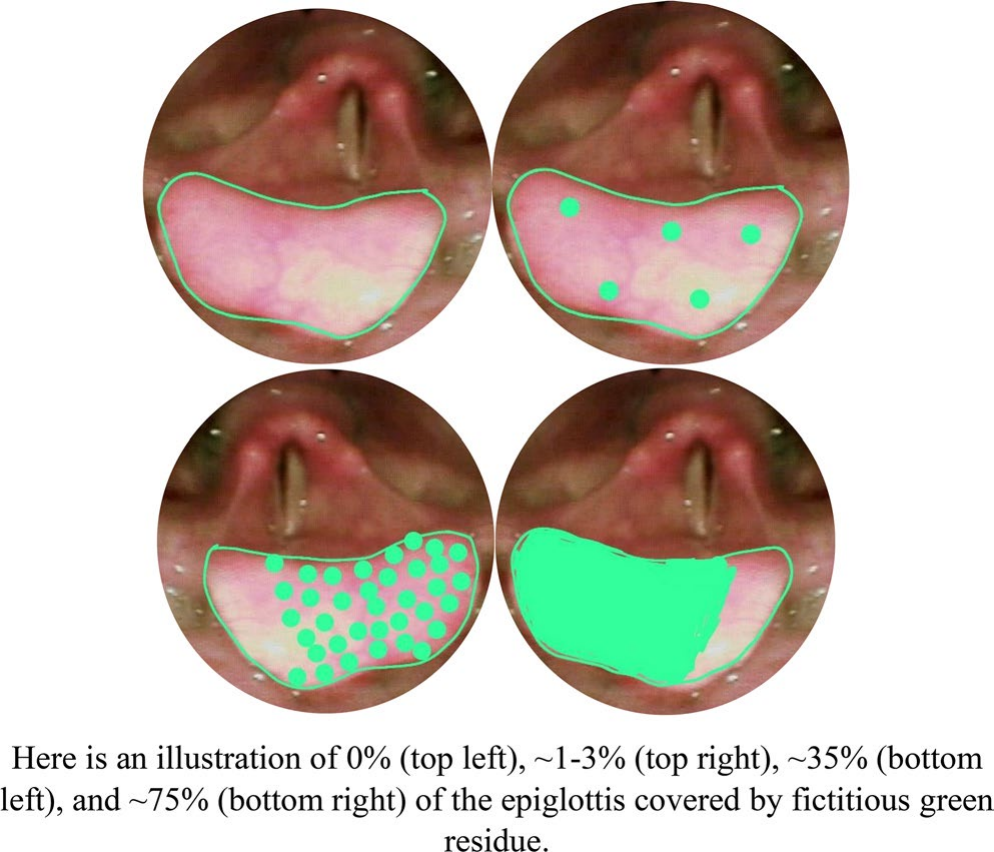
Examples of epiglottic residue covering. Here is an illustration of 0% (top left), ~1-3% (top right), ~35% (bottom left), and ~75% (bottom right) of the epiglottis covered by fictitious green residue.

Materials that could reasonably be interpreted as secretions or residue from a previous swallow should not be classified as new residue from the current swallow being evaluated. Residue ratings for the oropharynx demonstrated as an e.g. in Figure (6) and hypopharynx involve estimating the total amount of residue in these areas and expressing it as a percentage of how full the vallecula and/or piriform sinuses would be if all residues were collected. For the laryngeal surface of the epiglottis, see Figure (7), the laryngeal vestibule, and the vocal folds, residue ratings evaluate the percentage of the mucosal surface area that is covered with residue. This assessment includes both visible and non-visible areas for each anatomical landmark. Residue that is present on, but does not cross, the boundary between the epiglottis and laryngeal vestibule should be classified solely as residue on the epiglottis, with no consideration given to residue in the vestibule. Similarly, residue on, but not crossing, the border of the vocal folds and subglottis should be considered only as residue on the vocal folds, excluding any residue in the subglottic area [14].

## Data management and statistical analysis

The statistical package for the social sciences (SPSS) version 23.0 was used for data entry and analysis. The quantitative data were presented as mean, standard deviation and ranges when their distribution was parametric (normal), while non-normally distributed variables (non-parametric data) were presented as median with inter-quartile range (IQR). Also, qualitative variables were presented as numbers and percentages.

Assessment of the degree of reliability between observed readings and actual horizontal root fractures was evaluated by inter- & intra-class correlation coefficient (ICC) analysis (a two-way mixed model, an absolute agreement definition, and a single measure). The comparison between two periods for non-parametric data using the Wilcoxon signed-rank sum test. The confidence interval was set at 95%, and the accepted margin of error was 5%. The effect of the ES maneuver was quantified by calculating Cohen’s d for pre- and post-maneuver measurements from the VASE protocol.

## Results

**Table** 1: In our study’s demographic data, there was a notable male predominance, with males forming 70.6% (*n* = 24) of the sample, compared to females at 29.4% (*n* = 10). Regarding age distribution ranged from 39 to 90 years with a mean ± SD of 65.47 ± 13.30. Most patients had an ischemic stroke (70.6%, *n* = 24), while the remainder were diagnosed with hemorrhagic stroke (29.4%, *n* = 10). The A-EAT 10 questionnaire showed that 7 participants (20.6%) had a normal score, scoring below 3, while 27 participants (79.4%) scored 3 or higher. The scores on the A-EAT 10 ranged from 2 to 7, with an average score of 4.21 ± 1.65.

**Table 1:** Demographic data distribution

Demographic data	Total (*n* = 34)
Gender	
Male	24 (70.6%)
Female	10 (29.4%)
Age “years"	
Mean ± SD	65.47 ± 13.30
Range	39–90
Type of stroke	
Ischemic stroke	24 (70.6%)
Hemorrhagic stroke	10 (29.4%)
Arabic EAT-10	
EAT < 3	7 (20.6%)
EAT ≥ 3	27 (79.4%)
Mean ± SD	4.21 ± 1.65
Range	2–7
Swallowing	
Nasogastric tube	14(41.1)
Oral feeding	20(58.9)

The frequency of unsafe swallowing, indicated by a Penetration-Aspiration Scale (PAS) score exceeding 3, varied across bolus volumes and consistencies. The most pronounced effect was observed with thin fluids, where the number of affected participants escalated from 1 out of 34 (3%) at 3 cc to 4 out of 33 (12%) at 5 cc, and further to 11 out of 33 (33%) at 10 cc.(It should be noted that one patient was excluded from the 5 cc and 10 cc trials due to aspiration on the 3 cc bolus).For semisolids, 7 out of 34 participants (21%) presented with abnormal PAS scores, while for solids, the count was 4 out of 31 (13%), as three patients from the original cohort declined to attempt solid consistencies.

To validate the rating scores of the three observers, intra-rater (Reliability Within the same rater) and inter-rater reliability (Reliability between raters) analyses were performed.

## Intra-rater reliability

Regarding the Intra-Observer Reliability Coefficients (Tables 2 and 3), analysis of the intraclass correlation coefficient (ICC) showed statistically significant correlations, with most values exceeding 0.80 and many surpassing 0.90. This indicates a strong to near-perfect intra-observer reliability for the VASES protocol when assessing thin fluid volumes: (1 cc, 3 cc, 5 cc, 10 cc), semi-solids (a small spoon of yogurt), and solids (a small piece of bread). This elevated level of agreement was consistently observed across all evaluated parameters, including oropharyngeal residue, hypopharyngeal residue, laryngeal vestibule residue, vocal fold residue, epiglottic residue, subglottic residue, and penetration-aspiration scale (PAS) scores, with p-values of less than 0.05.

**Table 2:** Intra-observer reliability coefficients in 1CC, 3CC, and 5CC thin fluids

VASES Parameters	Intra-observer Reliability in 1CC	Intra-observer Reliability in 3CC	Intra-observer Reliability in 5CC
	ICC (95% CI)	ICC (95% CI)	ICC (95% CI)
Oropharyngeal residue			
Pre ES	0.823 (0.780–0.890)	0.825 (0.790–0.860)	0.805 (0.770–0.830)
Post ES	0.946 (0.900–0.980)	0.939 (0.910–0.960)	0.868 (0.840–0.890)
Hypopharyngeal residue			
Pre ES	0.798 (0.750–0.840)	0.804 (0.770–0.830)	0.916 (0.880–0.940)
Post ES	0.835 (0.790–0.880)	0.872 (0.840–0.900)	0.960 (0.930–0.980)
Vocal fold			
Pre ES	0.914 (0.870–0.950)	0.923 (0.890–0.950)	0.835 (0.800–0.860)
Post E	0.962 (0.930–0.990)	0.961 (0.930–0.980)	0.890 (0.860–0.920)
Laryngeal vestibule			
Pre ES	0.851 (0.810–0.900)	0.841 (0.810–0.870)	0.821 (0.790–0.850)
Post ES	0.879 (0.840–0.920)	0.896 (0.860–0.920)	0.929 (0.900–0.950)
Epiglottis			
Pre ES	0.807 (0.760–0.850)	0.820 (0.780–0.850)	0.798 (0.760–0.820)
Post ES	0.928 (0.890–0.960)	0.930 (0.900–0.950)	0.879 (0.850–0.900)
Sub glottic			
Pre ES	0.744 (0.700–0.790)	0.799 (0.760–0.830)	0.854 (0.820–0.880)
Post ES	0.886 (0.850–0.920)	0.883 (0.850–0.910)	0.915 (0.880–0.940)
PAS			
Pre ES	0.817 (0.780–0.850)	0.857 (0.820–0.890)	0.953 (0.920–0.980)
Post ES	0.904 (0.870–0.940)	0.912 (0.880–0.940)	0.810 (0.780–0.840)

using *ICC* intraclass correlation coefficient,; *PAS* penetration aspiration scale, *ES* effortful swallow

**Table 3:** Intra-observer reliability coefficients in 10CC thin fluids, Semi-solids, and solids

VASES parameters	Intra-observer Reliability in 10CC	Intra-observer Reliability in Semi-solids	Intra-observer Reliability in Solids
	ICC (95% CI)	ICC (95% CI)	ICC (95% CI)
Oropharyngeal residue			
Pre ES	0.924 (0.890–0.950)	0.883 (0.850–0.910)	0.921 (0.890–0.940)
Post ES	0.959 (0.930–0.980)	0.846 (0.810–0.870)	0.947 (0.920–0.960)
Hypopharyngeal residue			
Pre ES	0.836 (0.800–0.860)	0.913 (0.880–0.940)	0.829 (0.790–0.860)
Post ES	0.894 (0.860–0.920)	0.904 (0.870–0.930)	0.934 (0.900–0.960)
Vocal fold			
Pre ES	0.825 (0.790–0.850)	0.827 (0.790–0.860)	0.809 (0.770–0.840)
Post E	0.935 (0.900–0.960)	0.935 (0.900–0.960)	0.867 (0.830–0.890)
Laryngeal vestibule			
Pre ES	0.807 (0.770–0.830)	0.811 (0.770–0.840)	0.919 (0.880–0.940)
Post ES	0.882 (0.850–0.910)	0.865 (0.830–0.890)	0.954 (0.920–0.980)
Epiglottis			
Pre ES	0.856 (0.820–0.880)	0.918 (0.880–0.940)	0.837 (0.800–0.870)
Post ES	0.918 (0.880–0.940)	0.951 (0.920–0.970)	0.892 (0.860–0.920)
Sub glottic			
Pre ES	0.951 (0.920–0.970)	0.839 (0.800–0.870)	0.824 (0.790–0.850)
Post ES	0.814 (0.780–0.840)	0.896 (0.860–0.920)	0.936 (0.900–0.960)
PAS			
Pre ES	0.891 (0.860–0.920)	0.822 (0.780–0.850)	0.792 (0.750–0.820)
Post ES	0.850 (0.820–0.870)	0.928 (0.890–0.950)	0.877 (0.840–0.900)

using *ICC *Intraclass correlation coefficient,; *PAS *penetration aspiration scale, *ES *effortful swallow

## Inter-rater reliability 

Regarding the Inter-Observer Reliability Coefficients, excellent inter-rater reliability was observed among all three observers for the VASES across all volumes of thin liquids (1 cc, 3 cc, 5 cc, and 10 cc), as shown in Tables (4, 5, 6, 7), as well as for semi-solids (Table 8) and solids (Table 9), with a p-value of less than 0.05. This consistency was noted across all assessed parameters, including oropharyngeal, hypopharyngeal, laryngeal vestibule, vocal folds, epiglottic, and subglottic residue, in addition to penetration-aspiration scale scores.

**Table 4:** Inter-observer reliability coefficients in 1CC thin fluids

VASES parameters	Observer 1 & 2	Observer 1 & 3	Observer 2 & 3
	ICC (95% CI)	ICC (95% CI)	ICC (95% CI)
Oropharyngeal residue			
Pre ES	0.758(0.500–0.892.500.892)	1.000(1.000–1.000)	0.898 (0.662–0.965)
Post ES	1.000(1.000–1.000)	1.000(1.000–1.000)	1.000(1.000–1.000)
Hypopharyngeal residue			
Pre ES	0.893 (0.481–0.996)	0.819(0.640–0.909)	0.912 (0.845–1.000.845.000)
Post ES	1.000(1.000–1.000)	1.000(1.000–1.000)	1.000(1.000–1.000)
Vocal Fold			
Pre ES	1.000(1.000–1.000)	1.000(1.000–1.000)	1.000(1.000–1.000)
Post ES	1.000(1.000–1.000)	1.000(1.000–1.000)	1.000(1.000–1.000)
Laryngeal vestibule			
Pre ES	1.000(1.000–1.000)	1.000(1.000–1.000)	1.000(1.000–1.000)
Post ES	1.000(1.000–1.000)	1.000(1.000–1.000)	1.000(1.000–1.000)
Epiglottis			
Pre ES	1.000(1.000–1.000)	1.000(1.000–1.000)	1.000(1.000–1.000)
Post ES	1.000(1.000–1.000)	1.000(1.000–1.000)	1.000(1.000–1.000)
Sub Glottic			
Pre ES	1.000(1.000–1.000)	1.000(1.000–1.000)	1.000(1.000–1.000)
Post ES	1.000(1.000–1.000)	1.000(1.000–1.000)	1.000(1.000–1.000)
PAS			
Pre ES	1.000(1.000–1.000)	1.000(1.000–1.000)	1.000(1.000–1.000)
Post ES	1.000(1.000–1.000)	1.000(1.000–1.000)	1.000(1.000–1.000)

using *ICC *intraclass correlation coefficient, *ES *effortful swallow, *PAS *penetration aspiration scale

**Table 5:** Inter-observer reliability coefficients in 3CC thin fluids

VASES parameters	Observer 1 & 2	Observer 1 & 3	Observer 2 & 3
	ICC (95% CI)	ICC (95% CI)	ICC (95% CI)
Oropharyngeal residue			
Pre ES	0.876(0.716–0.942)	0.996(0.993–0.998)	0.891(0.741–0.950)
Post ES	0.869 (0.740–0.926)	0.953(0.906–0.977)	0.958 (0.619–0.991)
Hypopharyngeal residue			
Pre ES	0.814(0.603–0.910)	1.000(0.999–1.000.999.000)	0.817(0.610–0.911)
Post ES	0.846(0.693–0.923)	0.986(0.972–0.993)	0.863(0.726–0.931)
Vocal Fold			
Pre ES	0.750(0.503–0.875)	1.000(1.000–1.000)	0.750(0.503–0.875)
Post ES	1.000(1.000–1.000)	1.000(1.000–1.000)	1.000(1.000–1.000)
Laryngeal vestibule			
Pre ES	1.000(1.000–1.000)	1.000(1.000–1.000)	1.000(1.000–1.000)
Post ES	1.000(1.000–1.000)	1.000(1.000–1.000)	1.000(1.000–1.000)
Epiglottis			
Pre ES	1.000(1.000–1.000)	1.000(1.000–1.000)	1.000(1.000–1.000)
Post ES	1.000(1.000–1.000)	1.000(1.000–1.000)	1.000(1.000–1.000)
Sub Glottic			
Pre ES	0.983 (0.681–0.996)	1.000(1.000–1.000)	0.879 (0.788–0.927)
Post ES	1.000(1.000–1.000)	1.000(1.000–1.000)	1.000(1.000–1.000)
PAS			
Pre ES	1.000(1.000–1.000)	1.000(1.000–1.000)	1.000(1.000–1.000)
Post ES	1.000(1.000–1.000)	1.000(1.000–1.000)	1.000(1.000–1.000)

using *ICC *intraclass correlation coefficient, *ES *effortful swallow, *PAS *penetration aspiration scale

**Table 6:** Inter-Observer reliability coefficients in 5 cc thin fluids

VASES parameter	Observer 1 & 2	Observer 1 & 3		Observer 2 & 3
	ICC (95% CI)	ICC (95% CI)	ICC (95% CI)	
Oropharyngeal residue				
Pre ES	0.723(0.305–0.877)	0.997(0.995–0.999)	0.724(0.298–0.879)	
Post ES	0.774(0.466–0.896)	0.998(0.995–0.999)	0.778(0.492–0.897)	
Hypopharyngeal residue				
Pre ES	0.637(0.061–0.843)	0.993(0.985–0.996)	0.653(0.083–0.852)	
Post ES	0.650(0.207–0.837)	0.944(0.887–0.973)	0.776(0.227–0.914)	
Vocal Fold				
Pre ES	0.750(0.600–0.901.600.901)	0.989(0.979–0.995)	0.698 (0.562–0.765)	
Post ES	0.750(0.600–0.901.600.901)	1.000(1.000–1.000)	0.702 (0.589–0.817)	
Laryngeal vestibule				
Pre ES	0.935(0.869–0.968)	1.000(1.000–1.000)	0.935(0.869–0.968)	
Post ES	1.000(1.000–1.000)	1.000(1.000–1.000)	1.000(1.000–1.000)	
Epiglottis				
Pre ES	0.669(0.519–0.802)	1.000(1.000–1.000)	0.669(0.519–0.802)	
Post ES	1.000(1.000–1.000)	1.000(1.000–1.000)	1.000(1.000–1.000)	
Sub Glottic				
Pre ES	1.000(1.000–1.000)	1.000(1.000–1.000)	1.000(1.000–1.000)	
Post ES	1.000(1.000–1.000)	1.000(1.000–1.000)	1.000(1.000–1.000)	
PAS				
Pre ES	1.000(1.000–1.000)	1.000(1.000–1.000)	1.000(1.000–1.000)	
Post ES	0.990(0.980–0.995)	0.990(0.980–0.995)	1.000(1.000–1.000)	

using *ICC *intraclass correlation coefficient, *ES *effortful swallow, *PAS *penetration aspiration scale

**Table 7:** Inter-Observer reliability coefficients in 10CC thin fluids

VASES parameters	Observer 1 & 2	Observer 1 & 3		Observer 2 & 3
	ICC (95% CI)	ICC (95% CI)	ICC (95% CI)	
Oropharyngeal residue				
Pre ES	0.619(0.452–0.856)	0.987(0.974–0.993)	0.647(0.419–0.897)	
Post ES	0.716(0.118–0.887)	0.987(0.974–0.994)	0.746(0.193–0.899)	
Hypopharyngeal residue				
Pre ES	0.829(0.519–0.982)	0.971(0.935–0.986)	0.842(0.524–0.987)	
Post ES	0.617(0.514–0.817)	0.959(0.918–0.980)	0.601(0.501–0.802)	
Vocal Fold				
Pre ES	0.797(0.581–0.901)	0.996(0.992–0.998)	0.808(0.594–0.907)	
Post ES	0.852(0.702–0.927)	0.969(0.937–0.985)	0.911(0.817–0.957)	
Laryngeal vestibule				
Pre ES	0.627(0.245–0.818)	1.000(1.000–1.000)	0.627(0.245–0.818)	
Post ES	0.903(0.804–0.952)	1.000(1.000–1.000)	0.903(0.804–0.952)	
Epiglottis				
Pre ES	0.749(0.572–0.917)	0.882(0.764–0.942)	0.781(0.601–0.904)	
Post ES	1.000(1.000–1.000)	1.000(1.000–1.000)	1.000(1.000–1.000)	
Sub Glottic				
Pre ES	0.992(0.984–0.996)	0.997(0.994–0.999)	0.995(0.991–0.998)	
Post ES	0.972(0.943–0.986)	1.000(1.000–1.000)	0.972(0.943–0.986)	
PAS				
Pre ES	1.000(1.000–1.000)	1.000(1.000–1.000)	1.000(1.000–1.000)	
Post ES	0.990(0.980–0.995)	0.990(0.980–0.995)	1.000(1.000–1.000)	

using *ICC *intraclass correlation coefficient, *ES *effortful swallow, *PAS *penetration aspiration scale

**Table 8:** Inter-Observer reliability coefficients in semi-solids

VASES parameters	Observer 1 & 2	Observer 1 & 3	Observer 2 & 3
	ICC (95% CI)	ICC (95% CI)	ICC (95% CI)
Oropharyngeal residue			
Pre ES	0.885(0.698–0.949)	0.998(0.996–0.999)	0.877(0.670–0.946)
Post ES	0.695(0.194–0.868)	0.993(0.985–0.996)	0.670(0.229–0.848)
Hypopharyngeal residue			
Pre ES	0.853(0.630–0.934)	0.997(0.993–0.998)	0.848(0.632–0.931)
Post ES	0.879(0.760–0.939)	0.995(0.989–0.997)	0.899(0.799–0.949)
Vocal Fold			
Pre ES	0.979 (0.778–0.993)	1.000(1.000–1.000)	0.997 (0.538–0.999)
Post ES	0.719 (0.588–0.913)	1.000(1.000–1.000)	0.818 (0.619–0.905)
Laryngeal vestibule			
Pre ES	1.000(1.000–1.000)	1.000(1.000–1.000)	1.000(1.000–1.000)
Post ES	0.921(0.843–0.960)	1.000(1.000–1.000)	0.921(0.843–0.960)
Epiglottis			
Pre ES	0.896 (0.596–0.991)	0.705(0.413–0.852)	0.990 (0.936–0.996)
Post ES	0.691 (0.535–0.872)	0.795(0.592–0.897)	0.907 (0.772–0.953)
Sub Glottic			
Pre ES	0.999 (0.998–0.999)	0.977(0.955–0.989)	0.977 (0.893–0.991)
Post ES	0.733 (0.499–0.879)	0.997(0.995–0.999)	0.671 (0.133–0.844)
PAS			
Pre ES	1.000(1.000–1.000)	1.000(1.000–1.000)	1.000(1.000–1.000)
Post ES	0.995(0.990–0.998)	0.995(0.990–0.998)	1.000(1.000–1.000)

using *ICC *intraclass correlation coefficient, *ES *effortful swallow, *PAS *penetration aspiration scale

**Table 9:** Inter-Observer reliability coefficients in solids

VASES parameters	Observer 1 & 2	Observer 1 & 3	Observer 2 & 3
	ICC (95% CI)	ICC (95% CI)	ICC (95% CI)
Oropharyngeal residue			
Pre ES	0.981(0.960–0.991)	0.997(0.993–0.999)	0.985(0.961–0.993)
Post ES	0.985(0.960–0.994)	0.998(0.995–0.999)	0.989(0.973–0.995)
Hypopharyngeal residue			
Pre ES	0.949(0.887–0.976)	0.992(0.984–0.996)	0.942(0.863–0.973)
Post ES	0.923(0.836–0.963)	0.996(0.991–0.998)	0.914(0.810–0.960)
Vocal Fold			
Pre ES	0.983 (0.829–0.994)	1.000(1.000–1.000)	0.924 (0.421–0.893)
Post ES	0.988 (0.916–0.996)	1.000(1.000–1.000)	0.788 (0.619–0.902)
Laryngeal vestibule			
Pre ES	0.899 (0.796–0.999)	1.000(1.000–1.000)	0.848(0.619–0.995)
Post ES	0.729 (0.671–0.886)	1.000(1.000–1.000)	0.759(0.535–0.875)
Epiglottis			
Pre ES	1.000(1.000–1.000)	1.000(1.000–1.000)	1.000(1.000–1.000)
Post ES	0.759(0.502–0.918)	1.000(1.000–1.000)	0.659(0.487–0.815)
Sub Glottic			
Pre ES	0.849(0.533–0.947)	1.000(1.000–1.000)	0.749(0.617–0.872)
Post ES	0.759(0.588–0.918)	0.993(0.985–0.997)	0.857(0.714–0.984)
PAS			
Pre ES	1.000(1.000–1.000)	1.000(1.000–1.000)	1.000(1.000–1.000)
Post ES	1.000(1.000–1.000)	1.000(1.000–1.000)	1.000(1.000–1.000)

using *ICC, *intraclass correlation coefficient, *ES, *effortful swallow, *PAS *penetration aspiration scale

## Effect size of effortful swallow

The effect of the ES maneuver was quantified by calculating Cohen`s d for pre- and post-maneuver from the VASES protocol Tables (10, 11, 12, 13, 14 and 15), and it revealed these patterns among the three raters: For thin fluids, the effects of the ES maneuver were volume dependent. At the 1 cc volume, no significant differences in residue or PAS scores were observed across raters (*p* > 0.05; Table 10 ). A significant effect emerged at the 3 cc volume (Table 11), which demonstrated reductions in oropharyngeal (pre-ES d = 0.49, post-ES d = 0.78, *p* ≤ 0.041) and hypopharyngeal residue (pre-ES d = 0.51, post-ES d = 0.81, *p* ≤ 0.008), alongside improved PAS scores (d = 0.46, *p* = 0.021). These effects were strengthened at the 5 cc volume, showing greater reductions in oropharyngeal (pre-ES d = 0.59, post-ES d = 0.83) and hypopharyngeal residue (pre-ES d = 0.87, post-ES d = 1.28, *p* < 0.001), as well as in PAS scores (pre-ES d = 0.31, post-ES d = 0.38, *p* ≤ 0.005; Table 12). The largest residue reductions were observed at the 10 cc volume for both oropharyngeal (pre-ES d = 1.32, post-ES d = 1.57) and hypopharyngeal regions (pre-ES d = 1.39, post-ES d = 1.93, *p* < 0.001); however, PAS improvements at this volume only trended toward significance (*p* ≈ 0.06; Table 13). For semisolids, the ES maneuver yielded significant reductions in residue across both the oropharyngeal (pre-ES d = 0.92, post-ES d = 1.02) and hypopharyngeal regions (pre-ES d = 0.66, post-ES d = 0.88, *p* < 0.001). A statistically significant, though more modest, improvement was also observed in PAS scores (pre-ES d = 0.24, post-ES d = 0.27, *p* < 0.05). Beyond these quantitative measures, Rater 1 qualitatively reported additional improvements in vocal fold and epiglottis movement (Table 14). In contrast, for solid consistencies, the ES maneuver resulted in a significant decrease in residue (oropharyngeal: d = 0.33–0.38; hypopharyngeal: pre-ES d = 0.57, post-ES d = 0.58, *p* < 0.001), but no other measured parameters demonstrated significant change (Table 15).

**Table 10:** Effect sizes (Cohen’s d) for VASES parameters pre- and post-application of the ES maneuver with 1 cc thin fluids

VASES Parameters	Rater 1			Rater 2			Rater3		
	Pre ES	Post ES	*P*-value (Cohen’s d)	Pre ES	Post ES	*P*-value (Cohen’s d)	Pre ES	Post ES	*P*-value (Cohen’s d)
Oropharyngeal residue									
Median (IQR)	0(0–0)	0(0–0)	0.097 (0.455)	0(0–0)	0(0–0)	0.183 (1.73)	0(0–0)	0(0–0)	0.097 (0.455)
Range	0–15	0–0		0–13	0–0		0–15	0–0	
Hypopharyngeal residue									
Median (IQR)	0(0–0)	0(0–0)	0.106 (0.747)	0(0–0)	0(0–0)	0.122 (0.324)	0(0–0)	0(0–0)	0.719 (0.339)
Range	0–10	0–0		0–10	0–0		0–13	0–0	
Vocal Fold									
Median (IQR)	0(0–0)	0(0–0)	1.000 (0.000)	0(0–0)	0(0–0)	1.000 (0.000)	0(0–0)	0(0–0)	1.000 (0.000)
Range	0–0	0–0		0–0	0–0		0–0	0–0	
Laryngeal vestibule									
Median (IQR)	0(0–0)	0(0–0)	1.000 (0.000)	0(0–0)	0(0–0)	1.000 (0.000)	0(0–0)	0(0–0)	1.000 (0.000)
Range	0–0	0–0		0–0	0–0		0–0	0–0	
Epiglottis									
Median (IQR)	0(0–0)	0(0–0)	1.000 (0.000)	0(0–0)	0(0–0)	1.000 (0.000)	0(0–0)	0(0–0)	1.000 (0.000)
Range	0–0	0–0		0–0	0–0		0–0	0–0	
Sub Glottic									
Median (IQR)	0(0–0)	0(0–0)	1.000 (0.000)	0(0–0)	0(0–0)	1.000 (0.000)	0(0–0)	0(0–0)	1.000 (0.000)
Range	0–0	0–0		0–0	0–0		0–0	0–0	
PAS									
Median (IQR)	1(1–1)	1(1–1)	0.064 (0.470)	1(1–1)	1(1–1)	0.064 (0.470)	1(1–1)	1(1–1)	0.064 (0.470)
Range	0–2	0–1		0–2	0–1		0–2	0–1	

Using: Wilcoxon test, *ES *effortful swallow, *PAS *penetration aspiration scale, *IQR *Interquartile Range, *p*-value > 0.05 is insignificant; *p-value < 0.05 is significant; **p-value < 0.001 is highly significant

**Table 11:** Effect sizes (Cohen’s d) for VASES parameters pre- and post-application of the ES maneuver with 3 cc thin fluids

VASES parameters	Rater 1			Rater 2			Rater 3		
	Pre ES	Post ES	*P*-value (Cohen’s d)	Pre ES	Post ES	*P*-value (Cohen’s d)	Pre ES	Post ES	*P*-value (Cohen’s d)
Oropharyngeal residue									
Median (IQR)	2(0–6)	0(0–0)	< 0.001** (0.776)	0(0–3)	0(0–0)	0.041* (0.494)	2(0–5)	0(0–0)	0.002* (0.738)
Range	0–35	0–5		0–40	0–3		0–40	0–5	
Hypopharyngeal residue									
Median (IQR)	3(0–13)	0(0–1)	< 0.001** (0.813)	0(0–4)	0(0–0)	0.008* (0.514)	3(0–13)	0(0–3)	< 0.001** (0.812)
Range	0–25	0–10		0–30	0–15		0–25	0–10	
Vocal Fold									
Median (IQR)	0(0–0)	0(0–0)	0.325 (0.171)	0(0–0)	0(0–0)	0.325 (0.170)	0(0–0)	0(0–0)	0.325 (0.171)
Range	0–30	0–0		0–10	0–0		0–30	0–0	
Laryngeal vestibule									
Median (IQR)	0(0–0)	0(0–0)	1.000 (0.000)	0(0–0)	0(0–0)	1.000 (0.000)	0(0–0)	0(0–0)	1.000 (0.000)
Range	0–0	0–0		0–0	0–0		0–0	0–0	
Epiglottis									
Median (IQR)	0(0–0)	0(0–0)	1.000 (0.000)	0(0–0)	0(0–0)	1.000 (0.000)	0(0–0)	0(0–0)	1.000 (0.000)
Range	0–0	0–0		0–0	0–0		0–0	0–0	
Sub Glottic									
Median (IQR)	0(0–0)	0(0–0)	0.728 (0.045)	0(0–0)	0(0–0)	1.000 (0.000)	0(0–0)	0(0–0)	0.182 (0.235)
Range	0–15	0–10		0–0	0–0		0–15	0–0	
PAS									
Median (IQR)	1(1–2)	1(1–1)	0.021* (0.463)	1(1–2)	1(1–1)	0.021* (0.463)	1(1–2)	1(1–1)	0.021* (0.465)
Range	1–7	1–2		1–7	1–2		1–7	1–2	

Using: Wilcoxon test, *ES *Effortful Swallow, *PAS *penetration aspiration scale, *IQR *interquartile range, *p*-value > 0.05 is insignificant; *p-value < 0.05 is significant; **p-value < 0.001 is highly significant

**Table 12:** Effect sizes (Cohen’s d) for VASES parameters pre- and post-application of the ES maneuver with 5 cc thin fluids

VASES parameter	Rater 1			Rater 2			Rater 3		
	Pre ES	Post ES	*P*-value (Cohen’s d)	Pre ES	Post ES	*P*-value (Cohen’s d)	Pre ES	Post ES	*P*-value (Cohen’s d)
Oropharyngeal residue									
Median (IQR)	10(8–30)	3(0–10)	< 0.001** (0.818)	3(0–20))	0(0–5)	< 0.001** (0.588)	10(5–33)	3(0–10)	< 0.001** (0.83)
Range	0–55	0–30		0–50	0–30		0–60	0–30	
Hypopharyngeal residue									
Median (IQR)	25(13–33)	10(3–15)	< 0.001** (1.280)	15(3–25)	0(0–10)	< 0.001** (0.873)	25(10–35)	10(2–18)	< 0.001** (1.226)
Range	3–50	0–25		0–55	0–25		3–50	0–25	
Vocal Fold									
Median (IQR)	0(0–0)	0(0–0)	0.211 (0.172)	0(0–0)	0(0–0)	1.000 (0.000)	0(0–0)	0(0–0)	0.184 (0.151)
Range	0–25	0–10		0–20	0–20		0–20	0–10	
Laryngeal vestibule									
Median (IQR)	0(0–0)	0(0–0)	0.174 (0.240)	0(0–0)	0(0–0)	0.094 (0.300)	0(0–0)	0(0–0)	0.174 (0.240)
Range	0–5	0–0		0–5	0–0		0–5	0–0	
Epiglottis									
Median (IQR)	0(0–0)	0(0–0)	0.325 (0.240)	0(0–0)	0(0–0)	0.819 (0.080)	0(0–0)	0(0–0)	0.325 (0.170)
Range	0–3	0–0		0–2	0–0		0–3	0–0	
Sub Glottic									
Median (IQR)	0(0–0)	0(0–0)	1.000 (0.000)	0(0–0)	0(0–0)	1.000 (0.000)	0(0–0)	0(0–0)	1.000 (0.000)
Range	0–0	0–0		0–0	0–0		0–0	0–0	
PAS									
Median (IQR)	2(2–2)	2(2–2)	0.002* (0.380)	2(2–2)	2(2–2)	0.005* (0.314)	2(2–2)	2(2–2)	0.005* (0.315)
Range	2–6	1–5		2–6	1–6		2–6	1–6	

Using: Wilcoxon test, *ES *Effortful Swallow, *PAS *penetration aspiration scale, *IQR *Interquartile Range, *p*-value > 0.05 is insignificant; *p-value < 0.05 is significant; **p-value < 0.001 is highly significant

**Table 13:** Effect sizes (Cohen’s d) for VASES parameters pre- and post-application of the ES maneuver with 10 cc thin fluids

VASES parameters	Rater 1			Rater 2			Rater 3		
	Pre ES	Post ES	*P*-value (Cohen’s d)	Pre ES	Post ES	*P*-value (Cohen’s d)	Pre ES	Post ES	*P*-value (Cohen’s d)
Oropharyngeal residue									
Median (IQR)	35(25–55)	15(5–25)	< 0.001** (1.574)	30(8–35)	9(0–10)	< 0.001** (1.320)	30(25–55)	10(5–23)	< 0.001** (1.530)
Range	10–75	0–40		0–60	0–40		10–75	0–40	
Hypopharyngeal residue									
Median (IQR)	40(30–50)	20(10–23)	< 0.001** (1.875)	25(8–32)	3(0–13)	< 0.001** (1.390)	40(30–50)	20(10–25)	< 0.001** (1.930)
Range	5–70	0–40		0–55	0–35		5–75	0–40	
Vocal Fold									
Median (IQR)	0(0–10)	0(0–0)	0.004* (0.401)	0(0–0)	0(0–0)	0.050* (0.160)	0(0–10)	0(0–2)	0.005* (0.360)
Range	0–30	0–20		0–30	0–20		0–30	0–20	
Laryngeal vestibule									
Median (IQR)	0(0–3)	0(0–0)	0.005* (0.476)	0(0–0)	0(00)	0.161 (0.200)	0(0–3)	0(0–0)	0.005* (0.470)
Range	0–10	0–5		0–3	0–3		0–10	0–5	
Epiglottis									
Median (IQR)	0(0–0)	0(0–0)	0.160 (0.247)	0(0–0)	0(0–0)	0.317 (0.15)	0(0–0)	0(0–0)	0.083 (0.270)
Range	0–3	0–0		0–2	0–0		0–3	0–0	
Sub Glottic									
Median (IQR)	0(0–0)	0(0–0)	0.060 (0.201)	0(0–0)	0(0–0)	0.100 (0.230)	0(0–0)	0(0–0)	0.060 (0.220)
Range	0–60	0–30		0–60	0–30		0–60	0–30	
PAS									
Median (IQR)	2(2–5)	2(2–3)	0.062 (0.250)	2(2–5)	2(2–3)	0.064 (0.214)	2(2–5)	2(2–3)	0.064 (0.210)
Range	2–7	1–7		2–7	1–7		2–7	1–7	

Using: Wilcoxon test, *ES *effortful swallow, *PAS *penetration aspiration scale, *IQR *Interquartile Range, *p*-value > 0.05 is insignificant; *p-value < 0.05 is significant; **p-value < 0.001 is highly significant

**Table 14:** Effect sizes (Cohen’s d) for VASES parameters pre- and post-application of the ES maneuver with semi-solids

VASES parameters	Rater 1				Rater 2		Rater 3		
	Pre ES	Post ES	*P*-value (Cohen’s d)	Pre ES	Post ES	*P*-value (Cohen’s d)	Pre ES	Post ES	*P*-value (Cohen’s d)
Oropharyngeal residue									
Median (IQR)	23(10–63)	5(0–20)	< 0.001** (0.920)	15(4–40)	1(0–5)	< 0.001** (1.020)	23(10–64)	5(0–20)	< 0.001** (0.940)
Range	0–80	0–40		0–75	0–35		0–85	0–40	
Hypopharyngeal residue									
Median (IQR)	20(5–45)	5(0–15)	< 0.001** (0.880)	15(0–30)	3(0–15)	< 0.001** (0.660)	20(5–50)	5(0–11)	< 0.001** (0.880)
Range	0–55	0–30		0–50	0–35		0–60	0–30	
Vocal Fold									
Median (IQR)	0(0–0)	0(0–0)	0.050* (0.250)	0(0–0)	0(0–0)	0.829 (0.350)	0(0–0)	0(0–0)	0.050* (0.250)
Range	0–20	0–10		0–15	0–10		0–20	0–10	
Laryngeal Vestibule									
Median (IQR)	0(0–0)	0(0–0)	1.000 (0.000)	0(0–0)	0(0–0)	0.325 (0.090)	0(0–0)	0(0–0)	1.000 (0.000)
Range	0–3	0–3		0–3	0–3		0–3	0–3	
Epiglottis									
Median (IQR)	0(0–0)	0(0–0)	0.011* (0.520)	0(0–0)	0(0–0)	0.419 (0.770)	0(0–1)	0(0–0)	0.014* (0.490)
Range	0–10	0–3		0–13	0–3		0–15	0–3	
Sub Glottic									
Median (IQR)	0(0–0)	0(0–0)	0.047* (0.250)	0(0–0)	0(0–0)	0.719 (0.390)	0(0–0)	0(0–0)	0.086 (0.200)
Range	0–40	0–25		0–30	0–20		0–40	0–25	
PAS									
Median (IQR)	2(2–2)	2(1–2)	0.012* (0.270)	2(2–2)	2(1–2)	0.019* (0.240)	2(2–2)	2(1–2)	0.019* (0.240)
Range	1–7	1–7		1–7	1–7		1–7	1–7	

Using: Wilcoxon test, *ES *effortful swallow, *PAS *penetration aspiration scale, *IQR *Interquartile Range, *p*-value > 0.05 is insignificant; *p-value < 0.05 is significant; **p-value < 0.001 is highly significant

**Table 15:** Effect sizes (Cohen’s d) for VASES parameters pre- and post-application of the ES maneuver with solids

VASES parameters	Rater 1			Rater 2			Rater 3		
	Pre ES	Post ES	*P*-value (Cohen’s d)	Pre ES	Post ES	*P*-value (Cohen’s d)	Pre ES	Post ES	*P*-value (Cohen’s d)
Oropharyngeal residue									
Median (IQR)	3(0–30)	0(0–10)	< 0.001** (0.340)	0(0–20)	0(0–10)	0.002* (0.330)	3(0–25)	0(0–10)	< 0.001** (0.380)
Range	0–85	0–70		0–90	0–70		0–90	0–70	
Hypopharyngeal residue									
Median (IQR)	3(0–35)	0(0–10)	< 0.001** (0.580)	0(0–25)	0(0–10)	< 0.001** (0.570)	3(0–40)	0(0–10)	< 0.001** (0.570)
Range	0–75	0–35		0–60	0–40		0–80	0–40	
Vocal Fold									
Median (IQR)	0(0–0)	0(0–0)	0.087 (0.410)	0(0–0)	0(0–0)	0.291 (0.710)	0(0–0)	0(0–0)	0.087 (0.400)
Range	0–10	0–3		0–10	0–3		0–10	0–3	
Laryngeal vestibule									
Median (IQR)	0(0–0)	0(0–0)	0.161 (0.260)	0(0–0)	0(0–0)	0.098 (0.440)	0(0–0)	0(0–0)	0.161 (0.260)
Range	0–10	0–0		0–8	0–1		0–10	0–0	
Epiglottis									
Median (IQR)	0(0–0)	0(0–0)	1.000 (0.000)	0(0–0)	0(0–0)	0.558 (0.017)	0(0–0)	0(0–0)	1.000 (0.000)
Range	0–3	0–3		0–3	0–3		0–3	0–3	
Sub Glottic									
Median (IQR)	0(0–0)	0(0–0)	0.133 (0.240)	0(0–0)	0(0–0)	0.528 (0.32)	0(0–0)	0(0–0)	0.129 (0.210)
Range	0–25	0–25		0–20	0–20		0–25	0–25	
PAS									
Median (IQR)	2(1–2)	1(1–2)	0.086 (0.260)	2(1–2)	1(1–2)	0.086 (0.260)	2(1–2)	1(1–2)	0.086 (0.260)
Range	1–6	1–6		1–6	1–6		1–6	1–6	

Using: Wilcoxon test, *ES *effortful swallow, *PAS *penetration aspiration scale, *IQR *Interquartile Range, *p*-value > 0.05 is insignificant; *p-value < 0.05 is significant; **p-value < 0.001 is highly significant

## Discussion

While ES is widely used in clinical practice for the treatment of dysphagia, existing evidence lacks standardization in outcome measures. This study employed VASES, a tool designed to quantify both safety and efficiency under controlled volume/consistency conditions during FEES. Curtis et al. declared that the VASES protocol has high sensitivity in detecting swallow impairment, enabling clinicians to identify physiological deficits (such as vallecular or pyriform sinus residue) that may respond differently to rehabilitative maneuvers and tailor individualized treatment plans [19].

A recent systematic review by Jamil et al. highlights that many post-stroke dysphagia (PSD) intervention trials are hindered by small sample sizes and insufficient statistical power, which limit definitive conclusions regarding therapeutic efficacy [20]. In our study, the inclusion of a relatively large sample size of 34 post-stroke patients represents a notable improvement over many prior investigations. Larger studies enhance the robustness of statistical analyses and increase the generalizability of findings across diverse clinical populations. Although the present study focused on the immediate effects in a heterogeneous post-stroke cohort, we recognize that neuroplastic changes from swallowing maneuvers are typically driven by intensive, longer-term training [21]. The neurophysiological basis of the ES maneuver has been extensively studied, notably by Robbins et al., who demonstrated that ES increases submental muscle activation and pharyngeal pressure, facilitating improved bolus transit and airway closure. Their work elucidated the cortical and brainstem mechanisms involved in the voluntary modulation of swallowing effort, highlighting the potential of ES as a targeted rehabilitative exercise to induce neuroplastic changes [21]. Additionally, according to a longitudinal study by Malandraki et al., four weeks of ES training resulted in increased brain activation in the primary motor cortex and cerebellum, indicating that the technique can promote neuroplastic changes [22]. Accurately assessing oropharyngeal dysphagia is still a clinical challenge due to the complexity of swallowing physiology and the heterogeneity of patient presentations. While bedside clinical screening tools provide initial risk stratification, they often lack sensitivity and specificity, particularly in detecting silent aspiration and subtle pharyngeal residue [23]. The cutoff for an abnormal A-EAT-10 score was set at ≥ 3, based on the original validation by Belafsky et al. [24]. While other values have been proposed—such as a lower threshold of 2 to improve sensitivity Rofes et al. [25]. and a reported mean of 5.8 in asymptomatic Arabic individuals Farahat & Mosallam [18]. The Belafsky et al. [24] criterion was selected for its established balance in minimizing misclassification, thereby reducing the potential for both false-positive and false-negative outcomes. In our study, a discrepancy was observed in seven patients who, despite a normal A-EAT-10 score, exhibited penetration or aspiration (PAS score *≥* 3) on instrumental evaluation (FEES). Highlighting the limitation of relying solely on symptom questionnaires to rule out dysphagia. Matos et al. reported that most of the studies on swallowing rehabilitation used video fluoroscopy of swallowing [26]. Research specifically examining the efficacy of swallowing rehabilitation maneuvers in adult post-stroke dysphagia patients remains limited [17]. Furthermore, many techniques are implemented in conjunction with conventional therapies, which complicates the isolation and measurement of the efficacy of individual interventions [26]. Previous research evaluated the efficiency of ES to decrease residues, using video-fluoroscopy as in the study done by Donohue et al. [27], and another using brain activity by Malandraki et al. [22], but we assessed the efficiency of ES using the VASES protocol during FEES. FEES offers several advantages, including direct visualization of pharyngeal and laryngeal structures, the absence of radiation exposure, and applicability in diverse clinical settings, such as intensive care units [28]. Also, the comparatively low acquisition costs of FEES enhance its accessibility for both budget-constrained healthcare providers and populations in lower-income regions [5]. Despite its clinical utility, FEES interpretation has historically suffered from variability due to the lack of standardized rating protocols and clear anatomical landmarks for residue and airway invasion assessment [14]. This variability impedes both clinical decision-making and the comparability of research findings across centers. Curtis et al.. reported that the VASES protocol is a well-established framework that incorporates seven outcome measures to evaluate swallowing efficiency, with a particular emphasis on pharyngeal residue, as well as safety, concentrating on penetration and aspiration during individual swallows and bolus trials [14]. VASES was created to fill existing gaps and enhance the standardization and transparency of measuring pharyngeal residue, penetration, and aspiration, thereby aiding in the assessment of swallowing efficiency and safety as observed in FEES ratings [13]. Consequently, we employed the VASES protocol alongside FEES in our study. To validate the observers’ ratings, we assessed intra- and inter-rater reliability. The findings demonstrated strong agreement, thereby reducing the potential for subjective bias, and are consistent with prior VASES validation studies [14]. Following the implementation of ES, our data showed no statistically significant differences in swallowing safety or efficiency between the conditions with and without ES for the smallest thin fluid volume (1 cc), as most participants had normal swallow function (PAS scores 1–2) (Table 10**)**, so differences were unlikely to be observed. Furthermore, only 10 patients showed any residue, and in those cases, it was minimal (mere coating), further limiting the potential to detect an effect. These findings align with those of Steele et al. [29], Bahia and Lowell [17], who observed that minimal bolus volumes might not deliver enough stimulus to trigger measurable physiological changes during ES. In contrast, for larger fluid volumes (Tables 11, 12, and 13) (3 cc, 5 cc, and 10 cc) and thicker consistencies (semi-solids and solids) (Tables 14 and 15) ES significantly decreased oropharyngeal and hypopharyngeal residues (*p* < 0.001), with consistent ratings among observers. The reduction of residue is clinically meaningful as pharyngeal residue is a known risk factor for aspiration and subsequent pulmonary complications [30].

 Nativ-Zeltzer et al. further elucidated the physiological basis of these improvements, highlighting enhanced tongue base retraction and pharyngeal constriction as key mechanisms [31]. The significant decrease in PAS scores (*p* < 0.05) for 3 cc (Table 11) and 5 cc fluids (Table 12) and semi-solids (Table 14) shows improved airway protection with ES. Park et al. reported similar findings, proving that ES prolongs laryngeal vestibule closure, thereby reducing penetration and aspiration events in poststroke dysphagic patients [32]. Additionally, the observed reduction in vocal fold residue for 10 cc fluids and semi-solids during ES further supports enhanced airway clearance and safety, consistent with Steele et al. [33]. As regards no significant improvement in safety was noticed for solids (Table 15), we believe the primary reason is a fundamental lack of variance in the data for solid textures. As our cohort was broadly representative of post-stroke dysphagia, most participants had well-preserved swallowing safety for solids (PAS scores 1–2), creating a ceiling effect where ES could not demonstrate a further benefit. For the few patients who did have impaired safety with solids (*n* = 4 with PAS 5–8), the results were mixed: two patients showed marked improvement to a safe level (PAS < 2), while two did not. However, our trial was not powered to conduct meaningful subgroup analyses. Therefore, while a potential signal for efficacy in a specific severe subgroup may exist, our study design cannot confirm it.

The present study systematically evaluated the effects of the ES maneuver in post-stroke dysphagia using the VASES tool, demonstrating that both volume- and consistency-dependent improvements in swallowing safety (penetration-aspiration reduction) and efficiency (residue clearance), with the most pronounced benefits for ≥ 3 cc thin fluids and semisolids, and excellent inter-rater reliability of VASES across all tested parameters. These findings validate the clinical utility of ES as a targeted intervention, particularly in stroke populations where heterogeneous impairments demand precise tools, while establishing VASES as a standardized, reproducible tool for quantifying swallowing outcomes.

### Limitations

- Our primary aim was to evaluate the immediate effect within a broad post-stroke cohort, and our sample size was not powered to conduct meaningful subgroup analyses based on stroke characteristics or dysphagia severity. We fully acknowledge that this limits the generalizability of our findings. Future studies would benefit from stratifying participants by these key clinical metrics to determine their potential role as moderators of treatment response.
- We specifically aimed to investigate the isolated compensatory effect of ES on pharyngeal residue. To achieve this, we needed to control for confounding factors; thus, we excluded patients with significant cognitive deficits who would be unable to consistently comply with the procedure or for whom compensatory strategies are typically less suitable. We acknowledge that future studies are crucial to explore the efficacy and potential modifications of ES for patients with co-existing cognitive and physical limitations.
- Another limitation of this study is its focus on the immediate effects of a single session of the ES maneuver. While we documented significant compensatory benefits in reducing pharyngeal residue, our design does not allow us to determine whether these effects translate into long-term, rehabilitative gains with repeated practice. It is well-established that intensive, longer-term training of swallowing maneuvers can promote neuroplastic changes Robins et al. [21] and Malandraki et al. [22]. Therefore, the generalizability of our findings to the outcomes of a full therapeutic regimen is unknown. Future research employing longitudinal designs is necessary to investigate the cumulative and restorative effects of ES training over time.
- Successful patient performance of the maneuver was facilitated by the examiner’s demonstration and verbal cues during FEES. Nonetheless, quantitative assessment of performance accuracy was not feasible, as FEES lacks the capability for kinematic and or pressure measurement.

## Conclusion

The ES maneuver significantly improves pharyngeal clearance and airway protection for larger and more complex bolus volumes, supporting its role as an effective compensatory and rehabilitative strategy in post-stroke patients.

The VASES protocol offers a reliable and consistent way to interpret FEES results for post-stroke dysphagia.

## Data Availability

The data used and/or analyzed during the current study are available from the corresponding author on reasonable request.
